# Randomized controlled experimental study of hydrocortisone and D-cycloserine effects on fear extinction in PTSD

**DOI:** 10.1038/s41386-021-01222-z

**Published:** 2021-11-19

**Authors:** Sabra S. Inslicht, Andrea N. Niles, Thomas J. Metzler, Sa’ar L. Lipshitz, Christian Otte, Mohammed R. Milad, Scott P. Orr, Charles R. Marmar, Thomas C. Neylan

**Affiliations:** 1 University of California, San Francisco, San Francisco, CA USA; 2 San Francisco VA Health Care System, San Francisco, CA USA; 3 Charité-Universitätsmedizin, Berlin, Germany; 4 Grossman School of Medicine, New York University, New York, NY USA; 5 Massachusetts General Hospital, Charlestown, MA USA; 6 Harvard Medical School, Charlestown, MA USA

**Keywords:** Post-traumatic stress disorder, Fear conditioning

## Abstract

Fear extinction underlies prolonged exposure, one of the most well-studied treatments for posttraumatic stress disorder (PTSD). There has been increased interest in exploring pharmacological agents to enhance fear extinction learning in humans and their potential as adjuncts to PE. The objective of such adjuncts is to augment the clinical impact of PE on the durability and magnitude of symptom reduction. In this study, we examined whether hydrocortisone (HC), a corticosteroid, and D-Cycloserine (DCS), an N-methyl-D-aspartate receptor partial agonist, enhance fear extinction learning and consolidation in individuals with PTSD. In a double-blind placebo-controlled 3-group experimental design, 90 individuals with full or subsyndromal PTSD underwent fear conditioning with stimuli that were paired (CS+) or unpaired (CS−) with shock. Extinction learning occurred 72 h later and extinction retention was tested one week after extinction. HC 25 mg, DCS 50 mg or placebo was administered one hour prior to extinction learning. During extinction learning, the DCS and HC groups showed a reduced differential CS+/CS− skin conductance response (SCR) compared to placebo (*b* = −0.19, CI = −0.01 to −37, *p* = 0.042 and *b* = −0.25, CI = −08 to −0.43, *p* = 0.005, respectively). A nonsignificant trend for a lower differential CS+/CS− SCR in the DCS group, compared to placebo, (*b* = −0.25, CI = 0.04 to −0.55, *p* = 0.089) was observed at retention testing, one week later. A single dose of HC and DCS facilitated fear extinction learning in participants with PTSD symptoms. While clinical implications have yet to be determined, our findings suggest that glucocorticoids and NMDA agonists hold promise for facilitating extinction learning in PTSD.

## Introduction

Fear extinction is considered to be a critical component of prolonged exposure (PE), a well-studied treatment of choice for many patients with PTSD. In Pavlovian fear conditioning studies, individuals with PTSD were found to have an exaggerated fear response in some [1], but not all studies [2–4], impaired inhibition of conditioned fear [5], and deficits in fear extinction learning and recall, compared to healthy controls [2, 3, 6–8]. Given the central role of fear extinction in exposure-based treatments, a deficit in fear extinction learning and consolidation might explain why a significant proportion of PTSD patients remain symptomatic after treatment [9–12].

Cortisol is a primary stress hormone in humans that plays a role in fear extinction learning and memory consolidation. Cortisol is normally activated in response to stress and binds to glucocorticoid receptors resulting in negative feedback of the HPA axis, restraint of sympathetic nervous system arousal, and the eventual homeostatic return of the organism to baseline [13].

Pharmacological manipulations of cortisol and mechanisms related to learning and memory suggest that cortisol may have promise for enhancing extinction learning in PTSD. Numerous rodent studies have shown that corticosteroid administration facilitates fear extinction learning and memory [14–23]. Corticosteroids increase Ca^2+^ influx through L-type Ca^2+^ channels that affect long-term potentiation, a process important for neural plasticity (reviewed in [24]). Corticosteroids also inhibit glutamate uptake from the synaptic cleft resulting in prolonged activation of NMDA receptors that leads to MAPK signal cascades [25]. These processes are critical for fear extinction learning and memory formation. Thus, modulating the HPA axis may improve deficits in fear extinction learning and retention associated with PTSD.

More attention has focused on D-Cycloserine (DCS) as a drug that potentially may augment fear extinction due to its glutamatergic action on NMDA receptors [26]. Rodent studies have shown that DCS enhances fear extinction and reduces fear reinstatement when given before or soon after extinction training [27–29]. While DCS was shown to reduce self-reported arousal and amygdala activation between fear extinction learning and recall in imaging research [30], it failed to show an effect on psychophysiological measures in several studies [31, 32]. Because most of these DCS lab studies were conducted in healthy humans, there may have been floor effects due to subclinical impairment of fear extinction [31]. To date, there have been no studies examining DCS on laboratory measures of fear extinction in humans with PTSD.

This study examines the effects of hydrocortisone (HC), a synthetic form of cortisol, and DCS on fear extinction in a laboratory, differential fear-conditioning procedure in trauma-exposed individuals with PTSD symptoms. Human translational studies may be used to test whether candidate drugs act specifically by engaging mechanisms of fear extinction. The use of standardized stimuli, controlled laboratory environment and precision provided by objective physiological measurement confer an efficient approach for testing drug effects on fear learning mechanisms. The drugs found to affect extinction mechanisms may then be tested for use as adjuncts to exposure therapy.

We conducted a multi-day laboratory procedure in which fear conditioning was separated from extinction by 72 h to avoid potential interference with consolidation of conditioned learning. An extinction retention test was scheduled one week after the fear extinction session. This delay was chosen to provide a test of durability of extinction over time that would also allow for greater opportunity for spontaneous recovery, or the return of fear, and would provide evidence of longer duration of extinction consolidation. Orr and colleagues have previously shown that both extinction and conditioning memories were present one week after extinction [4]. Given evidence for a role of corticosteroids and DCS on processes involved in fear extinction and consolidation in rodent studies, we hypothesized that PTSD participants receiving HC or DCS would have greater fear extinction learning and retention, compared to those receiving placebo.

## Materials and methods

### Study design

**Fig. 1:**
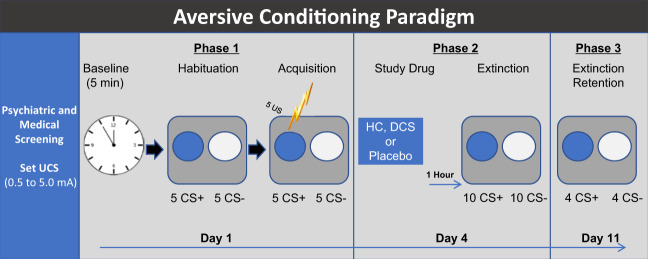
Timeline of study procedures. Psychophysiological testing occurred over 3 sessions. These included: Session 1: Habituation and fear conditioning. Session 2: Drug administration and fear extinction learning. Session 2 was scheduled 72 h following fear conditioning. A single dose of HC 25 mg, DCS 50 mg or placebo was administered 1 hr prior to extinction learning. Session 3: Extinction retention was tested 1 week after extinction learning.

The study design is a double-blind, randomized placebo-controlled experiment conducted over 4 study visits (Fig. 1) at the San Francisco VA Health Care System between January, 2009 and December, 2015. Procedures were approved by the University of California, San Francisco Institutional Review Board in accordance with the Declaration of Helsinki of 1975.

### Randomization

A randomization list was created by the biostatistician prior to study start and sent to the study physician and pharmacist. Drug conditions were randomized in a 1:1:1 ratio in blocks of 6, with separate lists for the male and female strata. Upon completion of Session 1, eligible participants were randomized to the study drug condition (Hydrocortisone, DCS, or placebo) by the physician. Study medication was dispensed to participants by a blinded study coordinator.

### Participants

Veterans and civilians were recruited from VA outpatient and community clinics, and internet advertisement. One-hundred and eleven participants, ages 18–65, were eligible by meeting full DSM-IV PTSD criteria or subsyndromal PTSD (i.e., CAPS score > 30 and meeting the A1, A2, B, E, and F clusters, and either the C or D clusters) for at least 3 months. Exclusion criteria included schizophrenia, bipolar disorder, alcohol dependence, drug abuse or dependence, seizure or neurological disorders, previous moderate or severe head injuries, current infectious illness, systemic illness affecting CNS function, or other conditions known to affect psychophysiological responses. Exclusionary medications included alpha and beta-adrenergics, antipsychotics, benzodiazepines, mood stabilizers, anticonvulsants, antihypertensives, sympathomimetics, anticholinergics, and steroids. Participants were alcohol- and drug-free during testing, as determined from self-report, urine drug screen, and breathalyzer.

A target sample size of 84 (28 per drug condition) was estimated from a power analysis for the detection of differences between each drug and placebo in differential skin conductance response (SCR; CS + vs. CS− trials; see Procedures below). Estimated power was 0.80 to detect standardized effects of d = 0.54 with an adjusted *p* value of 0.025 for each of the drug conditions, assuming a within-subjects correlation of r = 0.5 across repeated trials. The actual sample size was *N* = 90, with r = 0.2, yielding a minimum detectable effect of d = 0.42 at power = 0.80. One-hundred and six participants were randomized and 90 participants (*N* = 31 veterans and *N* = 59 civilians) completed all 3 psychophysiology sessions and were included in the analyses. A CONSORT diagram is presented in the Supplement.

### Measures

#### Clinical measures

PTSD diagnosis and symptom levels were determined by interview using the *Clinician Administered PTSD Scale (CAPS)* [33]. Other psychiatric and substance use disorders were assessed with the *Structured Clinical Interview for DSM-IV, version 2.0 (SCID I-NP)* [34].

#### Psychophysiological measures

Skin conductance level (SCL) was measured by a Coulbourn Isolated Skin Conductance coupler (S71–23) using a constant 0.5 V through 9-mm (sensor diameter) Sensor Medics Ag/AgCl electrodes that were placed 14 mm apart on the hypothenar surface of the participant’s non-dominant hand [35]. The SCL analog signal was digitized by a Coulbourn Lablinc Analog to Digital Converter (L25-12). A Microsoft Windows-based computer system was used for sampling and storing the digitized SCL signal and controlling stimulus presentations.

### Procedures

Following written informed consent, participants underwent a structured interview for psychiatric and medical history and provided blood and urine samples to assess exclusionary conditions and use of medications or drugs. Eligible participants then determined the shock level they found to be “highly annoying but not painful,” which would serve as the UCS in subsequent sessions scheduled one week later to avoid the possible influence of initial shock exposure on fear conditioning.

Session 1 was scheduled during the early follicular phase for cycling women to control for effects of reproductive hormone levels. All sessions were scheduled between 1:00 pm to 4:00 pm to minimize potential circadian effects. Participants were asked to abstain from caffeine, smoking, and eating for 1 h prior to the session and >1–2 alcohol beverages or illicit drugs for 72 h prior to participation.

Procedures and participant instructions were identical across sessions [6, 36]. Electrodes for SCL recording and those for administering the UCS were attached. The conditioned stimuli (CS+, paired with the UCS; CS−, not paired with the UCS) were 2 different colored computer generated 15.2 cm diameter circles, randomly selected for each participant and presented for 8 s on a 28.5 × 21.5 cm monitor positioned 1 m in front of the participant, with an intertrial interval of 20 ± 5 s. SCL was sampled at 1000 Hz beginning 2 s prior to CS onset and ending 6 s following CS offset. The UCS was a 500 ms electric pulse (0.5 to 5.0 mA) as previously determined by the participant, generated by a Coulbourn Transcutaneous Aversive Finger Stimulator (E13-22) via electrodes attached to the second and third fingers of the participant’s dominant hand. Participants were told that they “may or may not receive any electrical stimulation” prior to each session.

Session 1:

#### Habituation and fear conditioning

During Session 1 (Fig. 1) and following a 5-min baseline recording, participants were presented with 5 of each of the colored circles (to be CS+ and to be CS−) in the absence of the shock (UCS), constituting the habituation phase. This was followed by the fear conditioning phase, in which each presentation of the CS+ was followed by a 500 ms shock and the CS– was not. The CS+ and CS− were each presented 5 times. The CS+ and CS− stimuli in all phases of the experiment were presented in random order with no more than 2 consecutive presentations of the same stimulus type. After each session, participants were asked whether they could predict when the shock would occur and to identify the color of the CS+ to assess their contingency awareness.

Session 2:

#### Drug administration and fear extinction

The fear extinction phase was scheduled 72 h after fear conditioning, with study drug (HC 25 mg, DCS 50 mg, or placebo) administered 1 h prior to the fear extinction phase. The 25 mg HC dose was selected based on findings that 20–25 mg of HC was effective for facilitating memory, but that higher doses (i.e., 40 mg) were not [37, 38]. The 50 mg DCS dose was selected since it was the most commonly used dose in prior clinical trials and previously determined to be effective in enhancing extinction learning [39–41]. There was no difference found between studies using 50 mg and higher doses as shown in a secondary analyses of data from 21 clinical trials [39]. Drug and lactose placebo identically encapsulated pills were administered by a research coordinator who was blind to condition. The extinction learning phase followed identical instructions, set up procedures, and baseline recording as in the previous session, except that participants were presented with 10 non-reinforced presentations each of the CS+ and CS−.

Session 3:

#### Extinction retention

The extinction retention phase, conducted 1 week after extinction learning, consisted of 4 non-reinforced presentations each of the CS+ and CS−, following the same instructions and baseline recording. Upon completion, participants were debriefed, thanked, and reimbursed.

### Psychophysiological response scores

The SCR score for each CS interval was obtained by subtracting the mean SCL for the 2 s preceding CS onset from the peak during the 8 s CS interval [6, 36]. The UCR was obtained by subtracting the average SCL within 6–8 s following CS onset, from the maximum increase in SC level during the 0.5–6.5 s interval following CS offset (corresponding to the onset of the 0.5 s US). The SCR scores were signed-square-root transformed to normalize the skewed distribution. *Z* scores were then computed using within-participant means and standard deviations of normalized SCR scores across all conditions.

### Statistical analyses

Statistical analyses were conducted using linear mixed-effects modeling in Stata 16 [42]. Separate analyses were conducted for each phase, viz., fear conditioning, extinction learning, and extinction retention. The dependent measure used in all analyses was the SCR *z* score. Twenty-three outlier trials (0.4%), defined as *z* scores greater than 4 standard deviations from the mean, were winsorized (i.e., replaced with +/− 4). Following visual inspection of the standardized responses 4 standard deviations was chosen so as to balance the minimization of data loss with the prevention of extreme outliers’ undue influence on results. Each model included random intercepts for subjects and fixed effects for group (Placebo, HCS, DCS), stimulus type (CS−, CS+), and trials (5, habituation; 5, conditioning; 10, extinction learning; 4, extinction retention), and their interactions. Trials was modeled in 3 ways, as a continuous fixed effect, as a continuous random effect (i.e., with subject-specific slopes), and as a fixed categorical variable. The best fitting model was selected on the basis of likelihood tests or AIC fit criteria before conducting any statistical inferences. We report contrast results comparing differential SCRs (CS+ minus CS−) for HCS vs. Placebo and DCS vs. Placebo.

## Results

### Sample characteristics

Means, standard deviations, and results of ANOVA and Chi-Squared comparisons between groups for demographics and clinical characteristics are presented in Table 1. There were no significant differences between groups for age, sex, education, ethnicity/race, PTSD symptom severity, or use of psychiatric medications. Most participants (68/90) correctly identified the color that was paired with the shock, indicating explicit awareness of the CS-UCS contingency; there were no group differences.

**Table 1:** Descriptive statistics for sample.

	D-Cycloserine (*n* = 29)	Hydrocortisone (*n* = 31)	Placebo (*n* = 30)	F or χ^2^
	Mean (*SD*) Range or *N* (%)			
Age	40.1 (13.7) 20–61	34.3 (11.8) 18–63	39.8 (11.7) 22–64	2.08
Female, *N* (%)	17 (58.6)	19 (61.3)	14 (46.7)	1.48
Race, *N* (%)				4.69
White	18 (62.1)	20 (64.5)	22 (73.3)	
Black	5 (17.2)	3 (9.7)	5 (16.7)	
Asian	4 (13.8)	3 (9.7)	1 (3.3)	
Multiracial	2 (6.9)	5 (16.1)	2 (6.7)	
Hispanic or Latino Ethnicity	3 (10.3)	6 (19.4)	6 (20.0)	
Completed College, *N* (%)	16 (55.2)	13 (41.9)	10 (33.3)	2.90
Psychiatric Medications, *N* (%)	4 (13.8)	4 (12.9)	2 (6.7)	1.24
CAPS Score	58.1 (13.8) 32–80	53.93 (16.7) 30–100	60.0 (15.2) 34–90	1.26
UCS Level (0.5–5.0 mA)	2.46 (1.96)	2.54 (1.65)	2.89 (1.78)	0.49
Contingency Awareness	23 (79.3)	24 (77.4)	21 (70.0)	0.78
Habituation SCL (μS)	2.85 (4.18)	3.40 (2.77)	3.90 (3.32)	0.41
OR (μS)	0.31 (0.74)	0.25 (0.59)	0.20 (0.39)	0.17

*CAPS* Clinical Administered PTSD Scale, *UCS level* unconditioned stimulus level: the highest level of stimulation that participants self-selected to be “highly annoying but not painful”; Contingency Awareness: participants were asked whether they could predict when the shock would occur and to identify the color of the CS+; Habituation SCL: the average SCL for the 2-s pre-stimulus period across all CS+ trials during the Habituation phase; *OR* orienting response: SC response average to first presentation of the CS+ and CS− during the habituation phase.

### Baseline skin conductance and shock levels

As presented in Table 1, mean shock level, mean pre-stimulus SCL during habituation, and mean SC orienting response during the habituation phase did not differ between groups (*ps* > 0.60), nor were they associated with differential fear conditioning (*ps* > 0.48). Thus, differences between groups during extinction learning and retention are not likely to be attributable to differences in shock level selected, resting SCL, or SC orienting response magnitude.

#### Habituation and fear conditioning

Habituation and Conditioning phases were analyzed using a Group (Placebo, HCS, DCS) × Trials (5) × CS Type (CS+, CS−) random intercept mixed model with SCR *z* score as the dependent variable. Although we did not expect group differences during habituation or conditioning, or CS type differences during habituation, we tested these effects to confirm that there were no differences. The first CS+ and CS− trials of conditioning were dropped from analyses, because the UCS presentation occurs at the offset of CS+, so no conditioning can be observed until the second trial.

For habituation, there were no significant Group or CS Type effects, nor any significant interactions involving these factors (all p’s > 0.44). There was a significant main effect of Trials (*b* = −0.12, CI = −0.16, −0.08, *p* < 0.001), such that SCR to both CS+ and CS− significantly decreased over trials, indicating successful habituation to the CS stimuli.

During fear conditioning, there were no significant group differences for the differential SCR to CS+ vs. CS− trials (*p* = 0.53), no significant Group × Trials interaction (*p* = 0.36), and no Group × Trials × CS Type interaction (*p* = 0.12). There was a significant effect of CS+ vs. CS− (*b* = 0.68, CI = 0.52, 0.84, *p* < 0.001), indicating successful acquisition of fear responding.

#### Extinction learning effects

**Fig. 2:**
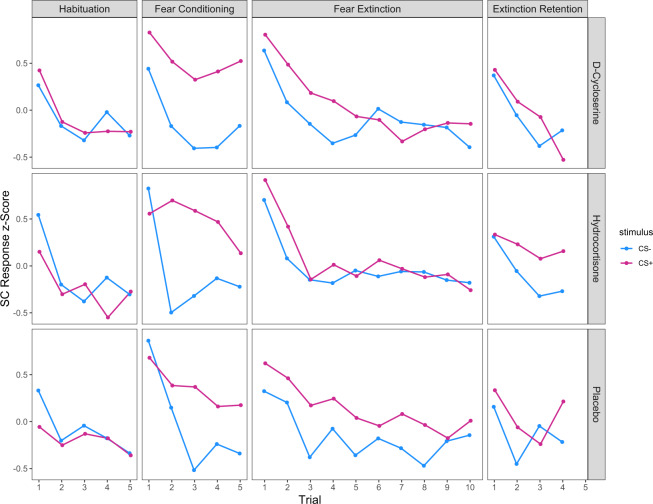
All study trials by group. Group mean skin conductance response scores for the conditioned stimulus (CS) intervals of CS+ and CS− trials during habituation, fear conditioning, extinction learning, and extinction retention. Data was converted to *z* scores to correct for variability in each participant’s range of responses.

**Fig. 3:**
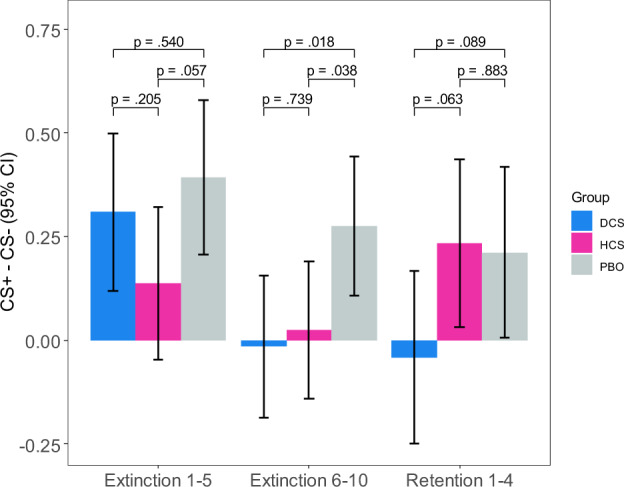
CS+/CS− difference for extinction learning and retention by group. CS+/CS− difference for early and late extinction (first 5 CS+ and 5 CS− trials and second 5 CS+ and 5 CS− trials) and retention (first 4 CS+ and 4 CS− trials) by group: Differential scores (CS+ minus CS−) for the conditioned stimulus (CS) intervals during the extinction learning and retention phases for each drug condition. Plotted values are marginal effects from the mixed models.

Extinction trials were analyzed using a random intercept mixed model similar to that for Habituation and Fear Conditioning, except that both linear and quadratic terms were included as random effects to model the nonlinear relationship of response over trials shown in Fig. 2. Extinction learning was evidenced by a CS Type × Trials interaction (χ^2^(1) = 4.75, *p* = 0.029). There was a Group × CS Type interaction (χ^2^(2) = 8.36, *p* = 0.015), which was attributable to smaller differences between SCRs to the CS+ and CS− in the 2 drug groups, compared to placebo (0.33 for Placebo vs. 0.15 for DCS (χ^2^(1) = 4.15, *p* = 0.042), and 0.08 for HC (χ^2^(1) = 7.82, *p* = 0.005). Group differences in extinction learning, averaged over early trials (trials1–5) and late trials (6–10), are shown in Fig. 3.

#### Extinction retention effects

Extinction retention was measured by the differential SCR (CS+ vs. CS−) over the 4 trials of the extinction retention phase, and analyzed using a Group × CS Type × Trials mixed model with random intercepts. While no main effects or interactions reached statistical significance, extinction learning appeared to be retained for the DCS group (X^2^ (1) = 2.89, *p* = .089) but not the HC group (X^2^ (1) = 0.02, *p* = .883) during the extinction retention phase. A post-hoc analysis comparing the last 5 trials of extinction learning to the 4 extinction retention trials demonstrated a greater differential response for HC at the retention phase compared to the extinction learning phase (X^2^ (1) = 3.88, *p* = 0.049) but not for DCS (X^2^ (1) = 0.37, *p* = 0.541) or Placebo (X^2^ (1) = 0.21, *p* = 0.648). The test for interaction between group (DCS, HCS, Placebo) and phase (extinction learning vs. extinction retention) was not significant (X^2^ (2) = 4.16, *p* = 0.125).

## Discussion

Our findings show that a single dose of HC or DCS facilitated fear extinction learning in trauma-exposed individuals with PTSD symptoms. Participants in both drug treatment arms showed evidence of enhanced fear extinction learning, compared to placebo. The pattern of results also shows a non-significant trend for greater extinction retention in the DCS but not HC group.

Although the results for consolidation of extinction were not supported by a statistically significant effect, findings support our hypotheses and data from prior studies showing that HPA axis- and NMDA activity enhance fear extinction learning, extending the current literature by showing that both DCS and HC modulate processes of fear extinction learning in patients with PTSD symptoms.

To our knowledge this is the first published study of DCS applied to a laboratory fear conditioning paradigm in a clinical PTSD population. Prior research has shown that individuals with PTSD have impaired extinction learning and retention [2, 6, 8]. As suggested by Davis, DCS may be less effective when glutamatergic receptors are already saturated [31, 43–45]. Our findings support the possibility that DCS may have a greater opportunity to show an effect in those with PTSD and potentially greater impairments in fear extinction.

Clinical care could be highly impacted if laboratory findings such as these lead to treatment innovation. Ideally, co-administration of a drug with exposure therapy, could facilitate extinction learning that is critical for effective treatment. In a randomized placebo-controlled pilot study, HC given 20 min prior to exposure therapy sessions was associated with a greater reduction in PTSD symptoms [46]. However, this finding was confounded by greater drop-out in the placebo group. A trial of the glucocorticoid dexamethasone paired with virtual reality exposure therapy for PTSD did not show evidence of the drug enhancing exposure treatment and was associated with greater drop out compared to placebo, possibly explained by increased reexperiencing symptoms [47]. In both studies, drug effects on fear extinction mechanisms could not be disentangled from effects on treatment engagement and retention.

Our HC findings for extinction learning are consistent with previous studies that found that cortisol administration enhanced fear extinction learning and retention in rodents [14–23] and healthy humans [48, 49]. HC also reduced PTSD symptoms in a study of traumatic memory reactivation [50]. The effects of HC on fear extinction learning and retention were previously shown to be mediated by the amygdala–hippocampus–vmPFC network [18], and can occur via potentiation of glutamatergic NMDA receptors [17, 51]. HC may also affect memory consolidation and retrieval processes through interactions with central noradrenergic systems [14, 52–54]. The effects of HC may be particularly important for individuals with PTSD; prior research has shown PTSD patients to have distinct HPA axis alterations, including increased CRH levels [55, 56] and increased glucocorticoid sensitivity that results in greater negative feedback of the HPA axis [57]. In the HC trial described above [46], greater responses to HC were seen in participants with higher lifetime PTSD symptoms and greater glucocorticoid sensitivity prior to treatment, suggesting that symptom severity, glucocorticoid receptor sensitivity, and the modulating chaperone protein FKBP5 may account for differences in response to glucocorticoids [58]. Although not measured in this study, future research should consider that individual differences in glucocorticoid signaling in PTSD may affect the potential benefit obtained from HC [59, 60].

While consistent with rodent studies demonstrating that DCS enhanced fear extinction [27–29], our results for DCS contrast with findings from several human laboratory studies of healthy participants that failed to show a benefit of DCS [31, 32, 44]. The different timing of DCS administration across studies may have impacted their results. Earlier human studies tended to initiate extinction learning on the same day as drug administration, soon after the acquisition of conditioned fear (e.g., [31, 32]). Because of the lack of separation between fear conditioning and extinction, it is possible that DCS acted to consolidate fear acquisition rather than improve extinction learning. Methodological differences in paradigms such as use of startle vs. shock as the UCS may also explain some differences.

While the present laboratory study focused on within-session extinction learning and retention 1 week later, psychotherapy treatment requires that extinction effects carry over between weekly sessions that are typically spread out over 8–12 weeks. While our results suggest possible differences in the persistence of DCS effects on extinction, compared to HC, the analysis comparing the three drug conditions was not significant.

A possible explanation for the lack of significant drug effect on extinction retention may be that our sample size is too small to detect a significant between-group effect. In addition, the extended time delay between end of extinction learning and the extinction retention test could have led to increased spontaneous recovery across all groups, thus masking any significant impact of drug treatment on the enhancement of extinction memory consolidation. Another possibility is that our primary outcome measure, SCR, might not be sufficiently sensitive to detect drug effects during the extinction retention phase. It is possible that DCS and/or HC might have impacted the neural correlates of fear extinction at the circuit level. In support of this, a recent fMRI study examining the effect of THC on fear extinction revealed a significant effect on the neural correlates of fear extinction retention 1 week after THC treatment without any significant impact of THC on SCR [61]. Future neuroimaging studies would be useful to assess the efficacy of HC and DCS to enhance the neural circuits of laboratory-based fear extinction in humans with PTSD.

While clinical trials of DCS in PTSD patients have also been mixed [62–67], a meta-analyses concluded that DCS had a small effect on improving symptoms following exposure treatment compared to placebo in patients with anxiety, obsessive-compulsive disorder and PTSD [68]. In the largest clinical trial of PTSD patients to date, DCS resulted in a greater reduction of cortisol and startle reactivity to virtual reality scenes, compared to alprazolam or placebo, but had no effect on PTSD symptom ratings [66]. It may be that physiological measures are more sensitive to the modest drug effects on specific biomarkers, which may not extend to changes in subjective PTSD symptoms. Small drug effects may have been overshadowed by variance associated with other potent features of PTSD not associated with fear conditioning such as guilt and shame, as well as therapist effects, treatment adherence, patient readiness, engagement, and the changes in cognitive appraisal and world view that can impact psychotherapy efficacy.

There are several limitations of our study to consider. We did not obtain measures of glucocorticoid receptor sensitivity or the modulating chaperone protein FKBP5. FKBP5 was previously found to be associated with impaired fear extinction and hyperarousal symptoms in humans [69]. An important next step will be to further interrogate the clinical relevance of these findings and determine who may be helped by adjunctive drug treatment and how it works. These questions may be examined with the addition of blood biomarker measures (e.g., FKBP5, glucorticoid receptor sensivity), and laboratory-based assessments of physiological functioning in clinical trials. The advent of novel drugs that can more clearly target specific aspects of glucocorticoid signaling will be an important future direction of research to better determine mechanisms of action of these drugs.

Despite the aforementioned limitations, our findings point to a biological signal of an effect of HC and DCS on fear extinction learning in PTSD as measured in the laboratory. Since individuals with PTSD were previously shown to have fear extinction deficits, HC and DCS could be particularly helpful in treating PTSD. While our findings provide initial evidence that adjunctive drugs augment extinction learning in a clinical PTSD sample, therapeutic efficacy of these drugs has yet to be determined.

## References

[1] Norrholm SD, Glover EM, Stevens JS, Fani N, Galatzer-Levy IR, Bradley B (2015). Fear load: the psychophysiological over-expression of fear as an intermediate phenotype associated with trauma reactions. Int J Psychophysiol.

[2] Milad MR, Orr SP, Lasko NB, Chang Y, Rauch SL, Pitman RK (2008). Presence and acquired origin of reduced recall for fear extinction in PTSD: results of a twin study. J Psychiatr Res.

[3] Peri T, Ben-Shakhar G, Orr SP, Shalev AY (2000). Psychophysiologic assessment of aversive conditioning in posttraumatic stress disorder. Biol Psychiatry.

[4] Orr SP, Milad MR, Metzger LJ, Lasko NB, Gilbertson MW, Pitman RK (2006). Effects of beta blockade, PTSD diagnosis, and explicit threat on the extinction and retention of an aversively conditioned response. Biol Psychol.

[5] Jovanovic T, Ressler KJ (2010). How the Neurocircuitry and Genetics of Fear Inhibtion may inform our understanding of PTSD. Am J Psychiatry.

[6] Orr SP, Metzger LJ, Lasko NB, Macklin ML, Peri T, Pitman RK (2000). De novo conditioning in trauma-exposed individuals with and without posttraumatic stress disorder. J Abnorm Psychol.

[7] Blechert J, Michael T, Vriends N, Margraf J, Wilhelm FH (2007). Fear conditioning in posttraumatic stress disorder: evidence for delayed extinction of autonomic, experiential, and behavioural responses. Behav Res Ther.

[8] Norrholm SD, Jovanovic T, Olin IW, Sands LA, Karapanou I, Bradley B (2011). Fear extinction in traumatized civilians with posttraumatic stress disorder: relation to symptom severity. Biol Psychiatry.

[9] Cusack K, Jonas DE, Forneris CA, Wines C, Sonis J, Middleton JC (2016). Psychological treatments for adults with posttraumatic stress disorder: a systematic review and meta-analysis. Clin Psychol Rev.

[10] Bradley R, Greene J, Russ E, Dutra L, Westen D (2005). A multidimensional meta-analysis of psychotherapy for PTSD. Am J Psychiatry.

[11] Steenkamp MM, Litz BT, Hoge CW, Marmar CR (2015). Psychotherapy for Military-Related PTSD: a review of randomized clinical trials. JAMA.

[12] Steenkamp MM, Litz BT, Marmar CR (2020). First-line psychotherapies for Military-Related PTSD. JAMA.

[13] de Kloet ER, Joels M, Holsboer F (2005). Stress and the brain: from adaptation to disease. Nat Rev Neurosci.

[14] Roozendaal B (2003). Systems mediating acute glucocorticoid effects on memory consolidation and retrieval. Prog Neuropsychopharmacol Biol Psychiatry.

[15] Korte SM (2001). Corticosteroids in relation to fear, anxiety and psychopathology. Neurosci Biobehav Rev.

[16] Schwabe L, Joels M, Roozendaal B, Wolf OT, Oitzl MS (2012). Stress effects on memory: an update and integration. Neurosci Biobehav Rev.

[17] Yang YL, Chao PK, Lu KT (2006). Systemic and intra-amygdala administration of glucocorticoid agonist and antagonist modulate extinction of conditioned fear. Neuropsychopharmacology.

[18] Merz CJ, Hamacher-Dang TC, Stark R, Wolf OT, Hermann A (2018). Neural underpinnings of cortisol effects on fear extinction. Neuropsychopharmacology.

[19] Michopoulos V, Norrholm SD, Stevens JS, Glover EM, Rothbaum BO, Gillespie CF (2017). Dexamethasone facilitates fear extinction and safety discrimination in PTSD: a placebo-controlled, double-blind study. Psychoneuroendocrinology.

[20] Cai WH, Blundell J, Han J, Greene RW, Powell CM (2006). Postreactivation glucocorticoids impair recall of established fear memory. J Neurosci.

[21] Barrett D, Gonzalez-Lima F (2004). Behavioral effects of metyrapone on Pavlovian extinction. Neurosci Lett.

[22] de Quervain DJ, Aerni A, Schelling G, Roozendaal B (2009). Glucocorticoids and the regulation of memory in health and disease. Front Neuroendocrinol.

[23] McGaugh JL, Roozendaal B (2002). Role of adrenal stress hormones in forming lasting memories in the brain. Curr Opin Neurobiol.

[24] Orsini CA, Maren S (2012). Neural and cellular mechanisms of fear and extinction memory formation. Neurosci Biobehav Rev.

[25] Lu KT, Walker DL, Davis M. Mitogen-activated protein kinase cascade in the basolateral nucleus of amygdala is involved in extinction of fear-potentiated startle. J Neurosci. 2001;21:RC162:1–RC62:5. 10.1523/JNEUROSCI.21-16-j0005.2001 PMC6763147 11473133

[26] Santini E, Muller RU, Quirk GJ (2001). Consolidation of extinction learning involves transfer from NMDA-independent to NMDA-dependent memory. J Neurosci.

[27] Walker DL, Ressler KJ, Lu KT, Davis M (2002). Facilitation of conditioned fear extinction by systemic administration or intra-amygdala infusions of D-cycloserine as assessed with fear-potentiated startle in rats. J Neurosci.

[28] Ledgerwood L, Richardson R, Cranney J (2004). D-cycloserine and the facilitation of extinction of conditioned fear: consequences for reinstatement. Behav Neurosci.

[29] Ledgerwood L, Richardson R, Cranney J (2003). Effects of D-cycloserine on extinction of conditioned freezing. Behav Neurosci.

[30] Ebrahimi C, Gechter J, Lueken U, Schlagenhauf F, Wittchen HU, Hamm AO (2020). Correction: augmenting extinction learning with D-cycloserine reduces return of fear: a randomized, placebo-controlled fMRI study. Neuropsychopharmacology.

[31] Guastella AJ, Lovibond PF, Dadds MR, Mitchell P, Richardson R (2007). A randomized controlled trial of the effect of D-cycloserine on extinction and fear conditioning in humans. Behav Res Ther.

[32] Klumpers F, Denys D, Kenemans JL, Grillon C, van der Aart J, Baas JM (2012). Testing the effects of Delta9-THC and D-cycloserine on extinction of conditioned fear in humans. J Psychopharmacol.

[33] Blake DD, Weathers FW, Nagy LM, Kaloupek DG, Gusman FD, Charney DS (1995). The development of a clinician-administered PTSD scale. J Trauma Stress.

[34] First MB, Spitzer RL, Gibbon M, Williams JBW. Structured clinical interview for DSM-IV Axis I disorders, research version, non-patient edition. (SCID-I/NP). Biometrics Research, New York State Psychiatric Institute: New York; 2002.

[35] Fowles DC, Christie MJ, Edelberg R, Grings WW, Lykken DT, Venables PH (1981). Committee report. Publication recommendations for electrodermal measurements. Psychophysiology.

[36] Inslicht SS, Metzler TJ, Garcia NM, Pineles SL, Milad MR, Orr SP (2013). Sex differences in fear conditioning in posttraumatic stress disorder. J Psychiatr Res.

[37] Het S, Ramlow G, Wolf OT (2005). A meta-analytic review of the effects of acute cortisol administration on human memory. Psychoneuroendocrinology.

[38] Abercrombie HC, Kalin NH, Thurow ME, Rosenkranz MA, Davidson RJ (2003). Cortisol variation in humans affects memory for emotionally laden and neutral information. Behav Neurosci.

[39] Rosenfield D, Smits JAJ, Hofmann SG, Mataix-Cols D, de la Cruz LF, Andersson E (2019). Changes in dosing and dose timing of D-cycloserine explain its apparent declining efficacy for augmenting exposure therapy for anxiety-related disorders: an individual participant-data meta-analysis. J Anxiety Disord.

[40] Ressler KJ, Rothbaum BO, Tannenbaum L, Anderson P, Graap K, Zimand E (2004). Cognitive enhancers as adjuncts to psychotherapy: use of D-cycloserine in phobic individuals to facilitate extinction of fear. Arch Gen Psychiatry.

[41] Hofmann SG, Meuret AE, Smits JA, Simon NM, Pollack MH, Eisenmenger K (2006). Augmentation of exposure therapy with D-cycloserine for social anxiety disorder. Arch Gen psychiatry.

[42] StataCorp. (StataCorp LLC, College Station, TX, 2019).

[43] Davis M, Ressler K, Rothbaum BO, Richardson R (2006). Effects of D-cycloserine on extinction: translation from preclinical to clinical work. Biol Psychiatry.

[44] Guastella AJ, Dadds MR, Lovibond PF, Mitchell P, Richardson R (2007). A randomized controlled trial of the effect of d-cycloserine on exposure therapy for spider fear. J Psychiatr Res.

[45] Myers KM, Davis M (2007). Mechanisms of fear extinction. Mol Psychiatry.

[46] Yehuda R, Bierer LM, Pratchett LC, Lehrner A, Koch EC, Van Manen JA (2015). Cortisol augmentation of a psychological treatment for warfighters with posttraumatic stress disorder: Randomized trial showing improved treatment retention and outcome. Psychoneuroendocrinology.

[47] Maples-Keller JL, Jovanovic T, Dunlop BW, Rauch S, Yasinski C, Michopoulos V (2019). When translational neuroscience fails in the clinic: Dexamethasone prior to virtual reality exposure therapy increases drop-out rates. J Anxiety Disord.

[48] de Quervain DJ, Margraf J (2008). Glucocorticoids for the treatment of post-traumatic stress disorder and phobias: a novel therapeutic approach. Eur J Pharm.

[49] de Quervain D, Schwabe L, Roozendaal B (2017). Stress, glucocorticoids and memory: implications for treating fear-related disorders. Nat Rev Neurosci.

[50] Surís A, North C, Adinoff B, Powell CM, Greene R (2010). Effects of exogenous glucocorticoid on combat-related PTSD symptoms. Ann Clin Psychiatry.

[51] Yang YL, Chao PK, Ro LS, Wo YY, Lu KT (2007). Glutamate NMDA receptors within the amygdala participate in the modulatory effect of glucocorticoids on extinction of conditioned fear in rats. Neuropsychopharmacology.

[52] Roozendaal B, Okuda S, Van der Zee EA, McGaugh JL (2006). Glucocorticoid enhancement of memory requires arousal-induced noradrenergic activation in the basolateral amygdala. Proc Natl Acad Sci USA.

[53] Nathan SV, Griffith QK, McReynolds JR, Hahn EL, Roozendaal B (2004). Basolateral amygdala interacts with other brain regions in regulating glucocorticoid effects on different memory functions. Ann N. Y Acad Sci.

[54] Quirarte GL, Roozendaal B, McGaugh JL (1997). Glucocorticoid enhancement of memory storage involves noradrenergic activation in the basolateral amygdala. Proc Natl Acad Sci USA.

[55] de Kloet CS, Vermetten E, Geuze E, Lentjes EG, Heijnen CJ, Stalla GK (2008). Elevated plasma corticotrophin-releasing hormone levels in veterans with posttraumatic stress disorder. Prog Brain Res.

[56] Baker DG, Ekhator NN, Kasckow JW, Dashevsky B, Horn PS, Bednarik L (2005). Higher levels of basal serial CSF cortisol in combat veterans with posttraumatic stress disorder. Am J Psychiatry.

[57] Yehuda R (2002). Current status of cortisol findings in post-traumatic stress disorder. Psychiatr Clin North Am.

[58] Binder EB (2009). The role of FKBP5, a co-chaperone of the glucocorticoid receptor in the pathogenesis and therapy of affective and anxiety disorders. Psychoneuroendocrinology.

[59] Kellner M, Yehuda R, Arlt J, Wiedemann K (2002). Longitudinal course of salivary cortisol in post-traumatic stress disorder. Acta Psychiatr Scand.

[60] Yehuda R, Golier J (2009). Is there a rationale for cortisol-based treatments for PTSD?. Expert Rev Neurother.

[61] Hammoud MZ, Peters C, Hatfield JRB, Gorka SM, Phan KL, Milad MR (2019). Influence of Delta9-tetrahydrocannabinol on long-term neural correlates of threat extinction memory retention in humans. Neuropsychopharmacology.

[62] Baker JF, Cates ME, Luthin DR (2017). D-cycloserine in the treatment of posttraumatic stress disorder. Ment Health Clin.

[63] de Kleine RA, Hendriks GJ, Kusters WJ, Broekman TG, van Minnen A (2012). A randomized placebo-controlled trial of D-cycloserine to enhance exposure therapy for posttraumatic stress disorder. Biol Psychiatry.

[64] Difede J, Cukor J, Wyka K, Olden M, Hoffman H, Lee FS (2014). D-cycloserine augmentation of exposure therapy for post-traumatic stress disorder: a pilot randomized clinical trial. Neuropsychopharmacology.

[65] Litz BT, Salters-Pedneault K, Steenkamp MM, Hermos JA, Bryant RA, Otto MW (2012). A randomized placebo-controlled trial of D-cycloserine and exposure therapy for posttraumatic stress disorder. J Psychiatr Res.

[66] Rothbaum BO, Price M, Jovanovic T, Norrholm SD, Gerardi M, Dunlop B (2014). A randomized, double-blind evaluation of D-cycloserine or alprazolam combined with virtual reality exposure therapy for posttraumatic stress disorder in Iraq and Afghanistan War veterans. Am J Psychiatry.

[67] Scheeringa MS, Weems CF (2014). Randomized placebo-controlled D-cycloserine with cognitive behavior therapy for pediatric posttraumatic stress. J Child Adolesc Psychopharmacol.

[68] Mataix-Cols D, Fernandez de la Cruz L, Monzani B, Rosenfield D, Andersson E, Perez-Vigil A (2017). D-cycloserine augmentation of exposure-based cognitive behavior therapy for anxiety, obsessive-compulsive, and posttraumatic stress disorders: a systematic review and meta-analysis of individual participant data. JAMA Psychiatry.

[69] Galatzer-Levy IR, Andero R, Sawamura T, Jovanovic T, Papini S, Ressler KJ (2017). A cross species study of heterogeneity in fear extinction learning in relation to FKBP5 variation and expression: Implications for the acute treatment of posttraumatic stress disorder. Neuropharmacology.

